# Supplementary material for: The Type IV Pilus of Plasmid TP114 Displays Adhesins Conferring Conjugation Specificity and Is Important for DNA Transfer in the Mouse Gut Microbiota
Source: Microbiol Spectr. 2022 Mar 16;10(2):e02303-21. doi: 10.1128/spectrum.02303-21 (PMC9045228; doi:10.1128/spectrum.02303-21)
Supplement: SUPPLEMENTAL FILE 1 — Supplemental material. Download Spectrum02303-21_suppmental_file.pdf, PDF file, 3.1 MB [file spectrum02303-21_suppmental_file.pdf]

Supplemental File 1

The type IVb pilus of conjugative plasmid TP114 is required for conjugation in the mouse gut microbiota.

Nancy Allard[1], Kevin Neil[1], Frédéric Grenier[1] and Sébastien Rodrigue[1]*

[1] Département de biologie, Faculté des sciences, Université de Sherbrooke, Sherbrooke, QC J1K 2R1, Canada

* Correspondence may be addressed to Sébastien Rodrigue. Tel: 1-819-821-8000 ext. 62939; Fax: 1-819-821-8049; Email: Sebastien.Rodrigue@USherbrooke.ca

This Supplemental Material includes:

Supplementary Tables S1 to S2

Supplementary Figures S1 to S6

Supplementary References

Supplementary Table S1. Strains and plasmids used in this study.

Strain or plasmid	Description[a]	Source (reference)
Escherichia coli		
EC100D*pir+*	F- *mcrA* Δ(*mrr-hsdRMS-mcrBC*) ϕ80*dlacZ*Δ*M15* Δ*lacX74 recA1 endA1 araD139* Δ(*ara, leu*)*7697 galU galK* λ– *rpsL nupG pir+* (DHFR)	#ECP09500 (Lucigen)
VB111	MG1655Nx^R , K-12 F- λ- *ilvG– rfb-50 rph-1*	Ceccarelli *et al.* 2008 (1)
VB112	MG1655Rf^R , K-12 F- λ- *ilvG– rfb-50 rph-1*	Ceccarelli *et al.* 2008 (1)
Nissle1917	Wild type probiotic strain	Brady *et al.* 2013 (2)
KN01Δ*dapA*	Nissle1917Δ*dapA*, Sp^R, Sm^R	Neil *et al.* 2020 (3)
KN03	Nissle1917, Sm^R, Tc^R	Neil *et al.* 2020 (3)
BW25113	F- Δ(*araD-araB*)*567*, Δ*lacZ4787*(::*rrnB-3*), λ-, *rph-1*, Δ(*rhaD-rhaB*)*568*, *hsdR514*	CGSC #7636
BW25113Δ*fliA*:: Kn^R	Keio mutant JW1907	Baba *et al.* 2006 (4)
BW25113Δ*fliA*	Keio mutant JW1907 without Kn^R	This study
CP9	O4 : K10, K54/96[†] : H5 : F13	Charles Dozois gift
J96	O4 : K- : H5 : F13	Charles Dozois gift
QT590		Charles Dozois gift
QT4378		Charles Dozois gift
CH138	O1 : H7	Charles Dozois gift
MT78	APEC O2 : K1	Charles Dozois gift
QT326		Charles Dozois gift
ABU83972	O rough : K5	Charles Dozois gift
RS218	O18 : K1 : H7	Charles Dozois gift
Ec1a	O1 : K1 : H7	Charles Dozois gift
JD-1		Charles Dozois gift
Res13-Lact-PEA02-11	Swine isolate O20 (100.0) : H12 (99.78)	Poulin-Laprade *et al.* 2021 (5)
Res13-Sevr-PEB15-13	Swine isolate O20 (100.0) : H12 (99.78)	Poulin-Laprade *et al.* 2021 (5)
Res13-Lact-ER13-10	Swine isolate O167 (99.37) : H26 (98.63)	Poulin-Laprade *et al.* 2021 (5)

Res13-Lact-PEA23-13	Swine isolate O8 (99.75) : H30 (99.94)	Poulin-Laprade *et al.* 2021 (5)
Res13-Fini-PEB16-01	Swine isolate O58 (100.0) : H21 (100.0)	Poulin-Laprade *et al.* 2021 (5)
Res13-Sevr-PEB04-11	Swine isolate O76 (100.0) : H7 (100.0)	Poulin-Laprade *et al.* 2021 (5)
Res13-Lact-PEC11-32	Swine isolate O8 (100.0) : H25 (100.0)	Poulin-Laprade *et al.* 2021 (5)
Res13-Lact-EC07-17	Swine isolate O-untypeable : H4 (100.0)	Poulin-Laprade *et al.* 2021 (5)
Res13-Croi-PEC15-20	Swine isolate O24 (100.0) : H4 (100.0)	Poulin-Laprade *et al.* 2021 (5)
Res13-Fini-PEC11-01	Swine isolate O24 (100.0) : H4 (100.0)	Poulin-Laprade *et al.* 2021 (5)
Res13-Fini-PER14-11	Swine isolate O39 (99.92) : H21 (99.86)	Poulin-Laprade *et al.* 2021 (5)
R21-R122-P3-j42c-fc-02	Swine isolate	Poulin-Laprade *et al.* 2021 (5)
R21-m228-j-4-fc-01	Swine isolate	Poulin-Laprade *et al.* 2021 (5)
**Plasmid**		
pACYC184	*oriV*$_{p15A}$, Tc^R, Cm^R	ATCC 37033
pBAD30	*oriV*$_{p15A}$, *bla* (Ap^R), *araC*, P$_{BAD}$	Guzman *et al.* 1995 (6)
pE-FLP	*oriV*$_{pSC101ts}$, *flp*, *bla* (Ap^R)	Addgene #45978
pKD3	*oriV*$_{R6K}$, FRT flanked Cm^R, Ap^R, template for one-step recombineering	Addgene #45604
pSB1C3	*oriV*$_{pMB1}$, *cat* (Cm^R), Biobricks	IGEM
pSIM6	*oriV*$_{pSC101ts}$, Lambda Red recombinase, *bla* (Ap^R)	Datta *et al.*, 2006 (7)
pRed	pBAD30::*mCherry*	Huguet *et al.* 2020 (8)
pRCI	pBAD30::*rci*	This study
pPilS	*oriV*$_{p15A}$, *cat* (Cm^R), *araC*, P$_{BAD}$, *pilS*	Neil *et al.* 2020 (3)
pPilVA	*oriV*$_{p15A}$, *cat* (Cm^R), *araC*, P$_{BAD}$, *pilVA*	This study
pNeonGreen	pACYC184ΔTc^R,P$_{N25}$ NeonGreen	This study
TP114	IncI2 conjugative plasmid, *aph*(3')-1 (Kn^R)	DSM-4246 (DSMZ)
TP114Δ*pilS::cat*	TP114 deletion mutant for the *pilS* gene, FRT flanked Cm^R	Neil *et al.* 2020 (3)
TP114Δ*pilS*	TP114 deletion mutant for the *pilS* gene, where FRT flanked Cm^R has been removed	Neil *et al.* 2020 (3)
TP114Δ*rci::cat*	TP114 deletion mutant for the *rci* gene, FRT flanked Cm^R	This study
TP114Δ*rci*	TP114Δ*rci::cat* where FRT flanked Cm^R has been removed	This study

TP114Δ*pilV::cat*	TP114 deletion mutant for the *pilV* gene, FRT flanked Cm^R	This study
TP114Δ*pilV*	TP114Δ*pilV::cat* where FRT flanked Cm^R has been removed	This study
TP114Δ*pilV*::FLAG-*cat*	TP114 deletion mutant for the variable 3'-end of *pilV* gene replaced by a FLAG-tag, FRT flanked Cm^R	This study
TP114Δshufflon::*pilVA-cat*	TP114 deletion mutant for the shufflon with *pilVA* fixed	This study
TP114Δshufflon::*pilVA'-cat*	TP114 deletion mutant for the shufflon with *pilVA'* fixed	This study
TP114Δshufflon::*pilVB-cat*	TP114 deletion mutant for the shufflon with *pilVB* fixed	This study
TP114Δshufflon::*pilVB'-cat*	TP114 deletion mutant for the shufflon with *pilVB'* fixed	This study
TP114Δshufflon::*pilVC-cat*	TP114 deletion mutant for the shufflon with *pilVC* fixed	This study
TP114Δshufflon::*pilVC'-cat*	TP114 deletion mutant for the shufflon with *pilVC'* fixed	This study
TP114Δshufflon::*pilVD-cat*	TP114 deletion mutant for the shufflon with *pilVD* fixed	This study
TP114Δshufflon::*pilVD'-cat*	TP114 deletion mutant for the shufflon with *pilVD'* fixed	This study
TP114*pilS*-S56C*	TP114 with cysteine knock-in *pilS* at position S56	This study
TP114*pilS*-T69C*	TP114 with cysteine knock-in *pilS* at position T69	This study
TP114*pilS*-T91C*	TP114 with cysteine knock-in *pilS* at position T91	This study
TP114*pilS*-T107C*	TP114 with cysteine knock-in *pilS* at position T107	This study
TP114*pilS*-S125C*	TP114 with cysteine knock-in *pilS* at position S125	This study
TP114*pilS*-S136C*	TP114 with cysteine knock-in *pilS* at position S136	This study
TP114*pilS*-S151C*	TP114 with cysteine knock-in *pilS* at position S151	This study
TP114*pilS*-T161C*	TP114 with cysteine knock-in *pilS* at position T161	This study
TP114*pilS*-T170C*	TP114 with cysteine knock-in *pilS* at position T170	This study
TP114*pilS*-S174C*	TP114 with cysteine knock-in *pilS* at position S174	This study

[a] Ap^R, ampicillin; Cm^R, chloramphenicol; Km^R, kanamycin; Nx^R, nalidixic acid; Rf^R, rifampicin; Sp^R, spectinomycin; Sm^R, streptomycin; Tc^R, tetracycline; DAP, diaminopimelic acid; ts, thermosensitive.

*The same mutations have been done in eB-TP114

† K10-positive strains also react with both K96 and K54 (which cross-react)

Supplementary Table S2: Oligonucleotides used in this study

Context	Name	Sequence[a, b] (5’-3’)	Template	Amplicon
pNeonGreen	oNG1-F	cattatgttccggatctgcatcgc	pACYC184	pACYC184ΔTc's backbone
	oNG1-R	cgcattaaagcttatcgatgataag		
	oNG2-F	catcgataagctttaatgcggcatgctcataaaaaattta	NeonGreen g-block	Neongreen
	oNG2-R	tgcagatccggaacataatgctaaatcgtatgccctatag		
pRCI	orci3-F	gcatgcaagcttggctgttttg	pBAD30	pBAD30's backbone
	orci3-R	ttttgcctcctagagctcgaattcgctagccc		
	orci4-F	ttttgggctagcgaattcgagctctaggaggcaaaaatgtttcagaaaatcaaaattcgc	TP114	*rci*
	orci4-R	tcttctctcatccgccaaaacagccaagcttgcatgcctaatttgttaatggacaaatca		
pPilVA	opilV21-F	tctccatacccgtttttttgggctagcgtaggaggcaaaaatgaaaaagacagataaagg	TP114Δshufflon::*pilVA-cat*	*pilVA*
	opilV21-R	ggcgagcggtatcagctcactcaaaggcggtaatacggttttaagtttggtatccaaaaa		
	opil1-F	ttttgcctcctacgctagcccaaaaaaacggg	pBAD30	pBAD30's backbone
	opil1-R	ctgtcagaccaagtttactc		
	opil2-F	aaccgtattaccgcctttggag	pSB1C3	*cat*
	opil2-R	tcaatctaaagtatatatgagtaaacttggtctgacaggtaaacttggtctgacagctcg		
TP114Δ*rci::cat*	orci1-F	caagaatctctaccttcccccctttttgtctggaggggattgggaattagccatggtcc	pKD3	*cat*
	orci1-R	taaaaaatcactctgtagttgttctatcataagtgaatcctgtgtaggctggagctgctt		
	orci2-R	gtgatattgcatttcgaagcaag	TP114	Screen *rci* deletion clones
	orci2-R	ccaggacagcatattcctcc		
TP114Δ*pilV::cat*	opilV1-F	ctcactcgtaccgggaatattattctgaggattaagagcaatgggaattagccatggtcc	pKD3	*cat*
	opilV1-R	taaaaaatcactctgtagttgttctatcataagtgaatccgtgtaggctggagctgcttc		
	opilV2-F	ccgttcctttgtggcggaat	TP114	Screen *pilV* deletion clones
	opilV2-R	ccaggacagcatattcctcc		
TP114Δ*pilV*::FLAG-*cat*	opilV19-F	tcaccatcacggcgactacaaagacgatgacgacaagtaaatgggaattagccatggtcc	pKD3	*cat*
	opilV1-R	taaaaaatcactctgtagttgttctatcataagtgaatccgtgtaggctggagctgcttc		
	opilV20-F	cagtacaggggcgatactttcgtgccaatccggtcggtggcatcaccatcaccatcacggcgactaca	*cat* PCR	FLAG-tag-*cat*
	opilV1-R	taaaaaatcactctgtagttgttctatcataagtgaatccgtgtaggctggagctgcttc		

TP114Δshufflon::*pilVA*-*cat*	opilV13-F	cagtacaggggcgatactttcgtgccaatccggtgtgtggagaacttccggttcctctaa	TP114	*pilVA*
	opilV13-R	ggaccatggctaattcccatttaagtttggtatccaaaaa		
	opilV14-F	tttttggataccaaacttaaatgggaattagccatggtcc	pKD3	*cat*
	opilV1-R	taaaaaatcactctgtagttgttctatcataagtgaatccgtgtaggctggagctgcttc		
TP114Δshufflon::*pilVA'*-*cat*	opilV11-F	cagtacaggggcgatactttcgtgccaatccggtgtgtggagggcattaggtggaaagct	TP114	*pilVA'*
	opilV11-R	ggaccatggctaattcccatttaattgagagttacacagg		
	opilV12-F	cctgtgtaactctcaattaaatgggaattagccatggtcc	pKD3	*cat*
	opilV1-R	taaaaaatcactctgtagttgttctatcataagtgaatccgtgtaggctggagctgcttc		
TP114Δshufflon::*pilVB*-*cat*	opilV17-F	cagtacaggggcgatactttcgtgccaatccggttcgtggaaatcaataggttcatgtgc	TP114	*pilVB*
	opilV17-R	ggaccatggctaattcccatttagcggaagcagtgaacag		
	opilV18-F	ctgttcactgcttccgctaaatgggaattagccatggtcc	pKD3	*cat*
	opilV1-R	taaaaaatcactctgtagttgttctatcataagtgaatccgtgtaggctggagctgcttc		
TP114Δshufflon::*pilVB'*-*cat*	opilV7-F	cagtacaggggcgatactttcgtgccaatccggtacgtggaaaaaaattggcgcaggtga	TP114	*pilVB'*
	opilV7-R	ggaccatggctaattcccattcactggcaaatggcgtaaa		
	opilV8-F	tttacgccatttgccagtgaatgggaattagccatggtcc	pKD3	*cat*
	opilV1-R	taaaaaatcactctgtagttgttctatcataagtgaatccgtgtaggctggagctgcttc		
TP114Δshufflon::*pilVC*-*cat*	opilV5-F	cagtacaggggcgatactttcgtgccaatccggtatctggacaacggcaaaagtgaactt	TP114	*pilVC*
	opilV5-R	ggaccatggctaattcccatttaaatcccagcaccaggaa		
	opilV6-F	ttcctggtgctgggatttaaatgggaattagccatggtcc	pKD3	*cat*
	opilV1-R	taaaaaatcactctgtagttgttctatcataagtgaatccgtgtaggctggagctgcttc		
TP114Δshufflon::*pilVC'*-*cat*	opilV3-F	cagtacaggggcgatactttcgtgccaatccggtcggtggtctggtggcaataaagtgaa	TP114	*pilVC'*
	opilV3-R	ggaccatggctaattcccatttaaccgaaggggcaacaat		
	opilV4-F	attgttgccccttcggttaaatgggaattagccatggtcc	pKD3	*cat*
	opilV1-R	taaaaaatcactctgtagttgttctatcataagtgaatccgtgtaggctggagctgcttc		
TP114Δshufflon::*pilVD*-*cat*	opilV15-F	cagtacaggggcgatactttcgtgccaatccggtacgtggcagaaaaatggcggcggtac	TP114	*pilVD*
	opilV15-R	ggaccatggctaattcccattcactgacacaatgcataag		
	opilV16-F	cttatgcattgtgtcagtgaatgggaattagccatggtcc	pKD3	*cat*
	opilV1-R	taaaaaatcactctgtagttgttctatcataagtgaatccgtgtaggctggagctgcttc		
TP114Δshufflon::*pilVD'*-*cat*	opilV9-F	cagtacaggggcgatactttcgtgccaatccggtacgtggagaagggcatcaggtagcac	TP114	*pilVD'*
	opilV9-R	ggaccatggctaattcccattcactggcaaaccacgatgt		

	opilV10-F	acatcgtggtttgccagtgaatgggaattagccatggtcc	pKD3	*cat*
	opilV1-R	taaaaaatcactctgtagttgttctatcataagtgaatccgtgtaggctggagctgcttc		
TP114*pilS*-S56C*	opilS4-F	tctattcaatggtgcaat**g**caacattcaatcatccaatga	TP114	*pilS*-S56C
	opilS-R	aataagcgcccgaaggcgcttatccgatgcacatgaaaaagtgtaggctggagctgcttc		
TP114*pilS*-T69C*	opilS5-F	atgaacaaaacaatgtcctg**tgt**gtgattgccaacatgaa	TP114	*pilS*-T69C
	opilS-R	aataagcgcccgaaggcgcttatccgatgcacatgaaaaagtgtaggctggagctgcttc		
TP114*pilS*-T91C*	opilS6-F	ctgacagtaactatattaagt**g**tctctacgcgcaaggatt	TP114	*pilS*-T91C
	opilS-R	aataagcgcccgaaggcgcttatccgatgcacatgaaaaagtgtaggctggagctgcttc		
TP114*pilS*-T107C*	opilS7-F	ccgatatgatcgcagatacat**g**tggcgcaagcgcgaaaaa	TP114	*pilS*-T107C
	opilS-R	aataagcgcccgaaggcgcttatccgatgcacatgaaaaagtgtaggctggagctgcttc		
TP114*pilS*-S125C*	opilS8-F	ccgttacaatcacaacctctt**g**tgataaatactccttcaa	TP114	*pilS*-S125C
	opilS-R	aataagcgcccgaaggcgcttatccgatgcacatgaaaaagtgtaggctggagctgcttc		
TP114*pilS*-S136C*	opilS9-F	ccttcaatgttgttgaggcc**t**gcgtaccgaagaaaaactg	TP114	*pilS*-S136C
	opilS-R	aataagcgcccgaaggcgcttatccgatgcacatgaaaaagtgtaggctggagctgcttc		
TP114*pilS*-S151C*	opilS10-F	caatggtaaacgccctgcgtt**g**ttccagcgcaatctccaa	TP114	*pilS*-S151C
	opilS-R	aataagcgcccgaaggcgcttatccgatgcacatgaaaaagtgtaggctggagctgcttc		
TP114*pilS*-T161C*	opilS11-F	caatctccaagattaacaact**g**ttccacatcaactgtcga	TP114	*pilS*-S161C
	opilS-R	aataagcgcccgaaggcgcttatccgatgcacatgaaaaagtgtaggctggagctgcttc		
TP114*pilS*-T170C*	opilS12-F	catcaactgtcgatgcggca**tg**tgtttgctcatctgacag	TP114	*pilS*-T170C
	opilS-R	aataagcgcccgaaggcgcttatccgatgcacatgaaaaagtgtaggctggagctgcttc		
TP114*pilS*-S174C*	opilS13-F	atgcggcaactgtttgctcat**g**tgacagcaatacgctgac	TP114	*pilS*-S174C
	opilS-R	aataagcgcccgaaggcgcttatccgatgcacatgaaaaagtgtaggctggagctgcttc		
Shufflon experiment	oSh-F	acactctttccctacacgacgctcttccgatctcagtacaggggcgatactttcgtgcc	TP114	
	oSh-R	gtgactggagttcagacgtgtgctcttccgatct		3'-end *pilV*

*The same mutations were also introduced in eB-TP114

[a]Bold nucleotides represent the mutations introduced

[b]Underline represent binding nucleotides

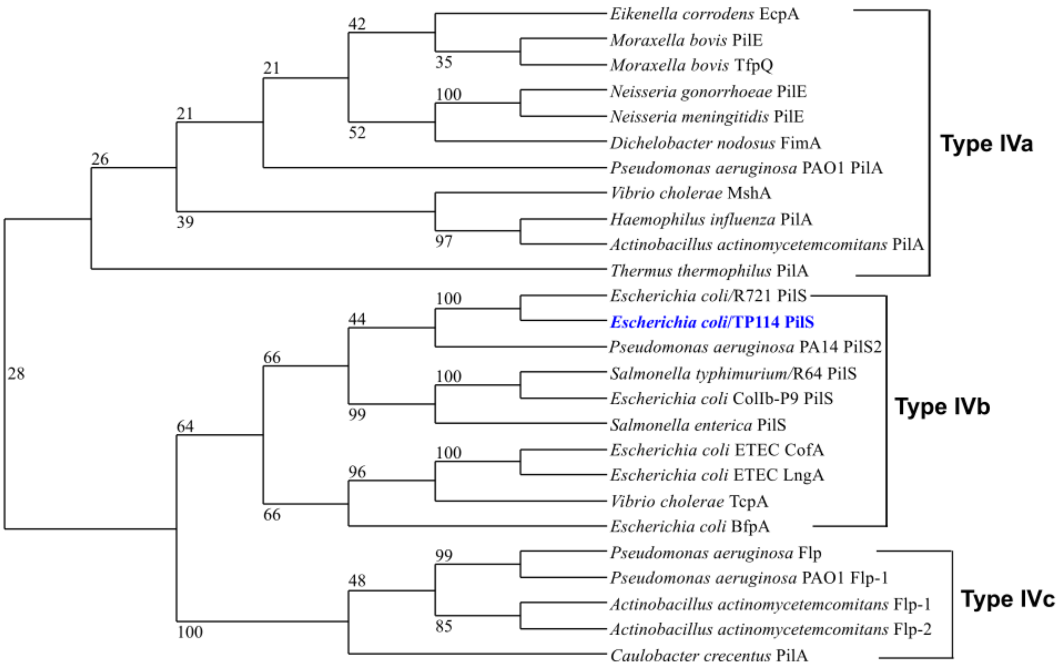

Supplementary Figure S1. The PilS protein forming the T4P of conjugative plasmid TP114 is part of the IVb family. Phylogenetic tree showing the relationship among major pilin amino acid sequences of selected bacterial species. The analyzed pilins are partitioned into three distinct classes IVa, IVb and IVc based on their primary sequences. The tree was generated by maximum likelihood analysis in SeaView 5.0.4 software. Bootstrap analysis was performed on 1000 trees and the values are provided on the branch nodes. The bacterial species and accession numbers of the amino acid sequences used for the alignment are listed in Supplementary Table S3. TP114 PilS is highlighted in blue.

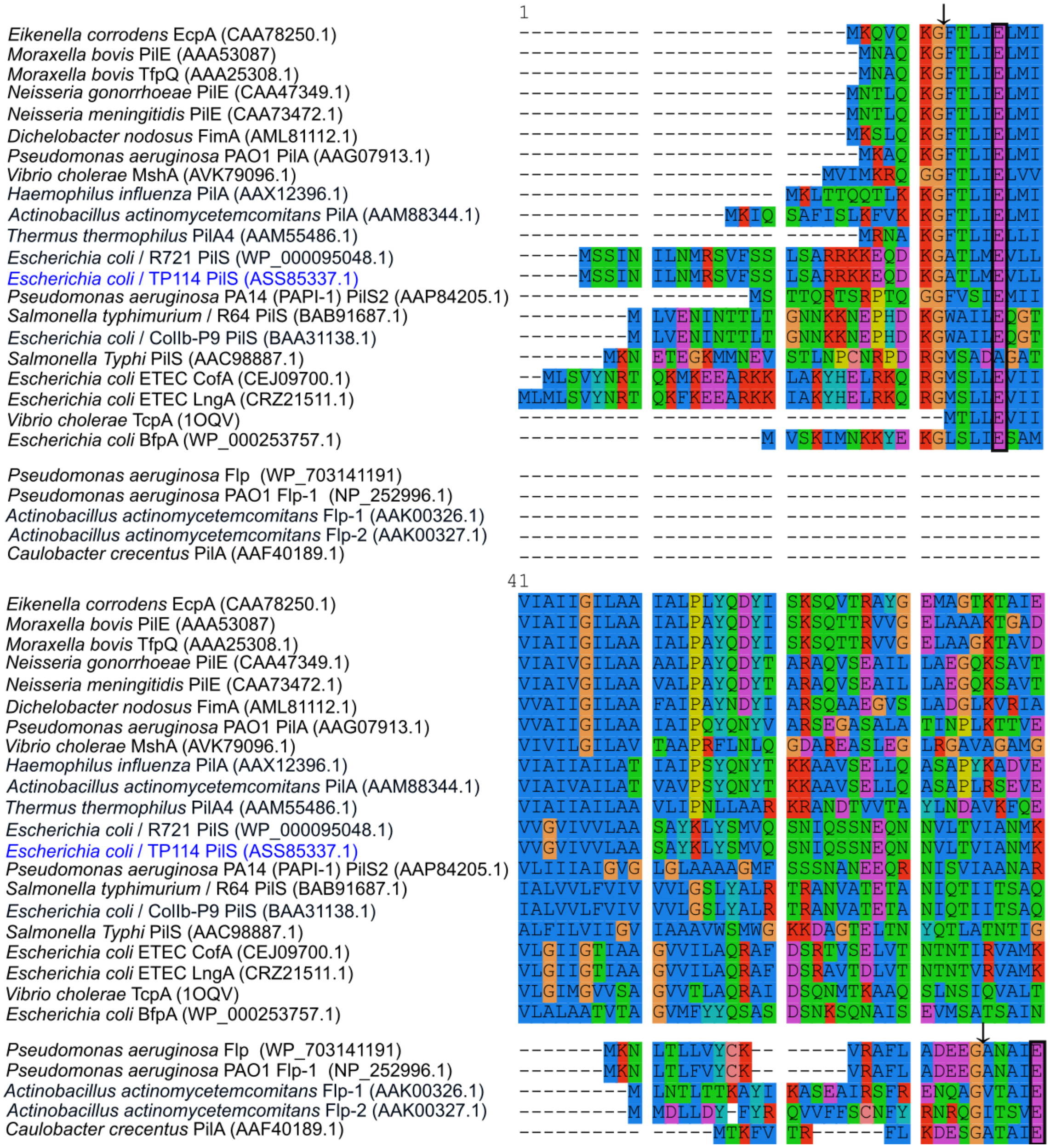
1
Eikenella corrodens EcpA (CAA78250.1) ---------- ---------- -----MKQVQ KGFTLIELMI
Moraxella bovis PilE (AAA53087) ---------- ---------- ------MNAQ KGFTLIELMI
Moraxella bovis TfpQ (AAA25308.1) ---------- ---------- ------MNAQ KGFTLIELMI
Neisseria gonorrhoeae PilE (CAA47349.1) ---------- ---------- -----MNTLQ KGFTLIELMI
Neisseria meningitidis PilE (CAA73472.1) ---------- ---------- -----MNTLQ KGFTLIELMI
Dichelobacter nodosus FimA (AML81112.1) ---------- ---------- -----MKSLQ KGFTLIELMI
Pseudomonas aeruginosa PAO1 PilA (AAG07913.1) ---------- ---------- ------MKAQ KGFTLIELMI
Vibrio cholerae MshA (AVK79096.1) ---------- ---------- ---MVIMKRQ GGFTLIELVV
Haemophilus influenza PilA (AAX12396.1) ---------- ---------- MKLTTQQTLK KGFTLIELMI
Actinobacillus actinomycetemcomitans PilA (AAM88344.1) ---------- ------MKIQ SAFISLKFVK KGFTLIELMI
Thermus thermophilus PilA4 (AAM55486.1) ---------- ---------- ------MRNA KGFTLIELLI
Escherichia coli / R721 PilS (WP_000095048.1) -----MSSIN ILNMRSVFSS LSARRKKEQD KGATLMEVLL
Escherichia coli / TP114 PilS (ASS85337.1) -----MSSIN ILNMRSVFSS LSARRKKEQD KGATLMEVLL
Pseudomonas aeruginosa PA14 (PAPI-1) PilS2 (AAP84205.1) ---------- --------MS TTQRTSRPTQ GGFVSIEMII
Salmonella typhimurium / R64 PilS (BAB91687.1) ---------M LVENINTTLT GNNKKNEPHD KGWAILEQGT
Escherichia coli / Collb-P9 PilS (BAA31138.1) ---------M LVENINTTLT GNNKKNEPHD KGWAILEQGT
Salmonella Typhi PilS (AAC98887.1) -------MKN ETEGKMMNEV STLNPCNRPD RGMSADAGAT
Escherichia coli ETEC CofA (CEJ09700.1) --MLSVYNRT QKMKEEARKK LAKYHELRKQ RGMSLLEVII
Escherichia coli ETEC LngA (CRZ21511.1) MLMLSVYNRT QKFKEEARKK IAKYHELRKQ RGMSLLEVII
Vibrio cholerae TcpA (1OQV) ---------- ---------- ---------- --MTLLEVII
Escherichia coli BfpA (WP_000253757.1) ---------- ---------M VSKIMNKKYE KGLSLIESAM
Pseudomonas aeruginosa Flp (WP_703141191) ---------- ---------- ---------- ----------
Pseudomonas aeruginosa PAO1 Flp-1 (NP_252996.1) ---------- ---------- ---------- ----------
Actinobacillus actinomycetemcomitans Flp-1 (AAK00326.1) ---------- ---------- ---------- ----------
Actinobacillus actinomycetemcomitans Flp-2 (AAK00327.1) ---------- ---------- ---------- ----------
Caulobacter crecentus PilA (AAF40189.1) ---------- ---------- ---------- ----------
41
Eikenella corrodens EcpA (CAA78250.1) VIAIIGILAA IALPLYQDYI SKSQVTRAYG EMAGTKTAIE
Moraxella bovis PilE (AAA53087) VIAIIGILAA IALPAYQDYI SKSQTTRVVG ELAAAKTGAD
Moraxella bovis TfpQ (AAA25308.1) VIAIIGILAA IALPAYQDYI SKSQTTRVVG ELAAGKTAVD
Neisseria gonorrhoeae PilE (CAA47349.1) VIAIVGILAA AALPAYQDYT ARAQVSEAIL LAEGQKSAVT
Neisseria meningitidis PilE (CAA73472.1) VIAIVGILAA VALPAYQDYT ARAQVSEAIL LAEGQKSAVT
Dichelobacter nodosus FimA (AML81112.1) VVAIIGILAA FAIPAYNDYI ARSQAAEGVS LADGLKVRIA
Pseudomonas aeruginosa PAO1 PilA (AAG07913.1) VVAIIGILAA IAIPQYQNYV ARSEGASALA TINPLKTTVE
Vibrio cholerae MshA (AVK79096.1) VIVILGILAV TAAPRFLNLQ GDAREASLEG LRGAVAGAMG
Haemophilus influenza PilA (AAX12396.1) VIAIIAILAT IAIPSYQNYT KKAAVSELLQ ASAPYKADVE
Actinobacillus actinomycetemcomitans PilA (AAM88344.1) VIAIVAILAT VAVPSYQNYT KKAAVSELLQ ASAPLRSEVE
Thermus thermophilus PilA4 (AAM55486.1) VIAIIAILAA VLIPNLLAAR KRANDTVVTA YLNDAVKFQE
Escherichia coli / R721 PilS (WP_000095048.1) VVGVIVVLAA SAYKLYSMVQ SNIQSSNEQN NVLTVIANMK
Escherichia coli / TP114 PilS (ASS85337.1) VVGVIVVLAA SAYKLYSMVQ SNIQSSNEQN NVLTVIANMK
Pseudomonas aeruginosa PA14 (PAPI-1) PilS2 (AAP84205.1) VLIIIAIGVG LGLAAAAGMF SSSNANEEQR NISVIAANAR
Salmonella typhimurium / R64 PilS (BAB91687.1) IALVVLFVIV VVLGSLYALR TRANVATETA NIQTIITSAQ
Escherichia coli / CollB-P9 PilS (BAA31138.1) IALVVLFVIV VVLGSLYALR TRANVATETA NIQTIITSAQ
Salmonella Typhi PilS (AAC98887.1) ALFILVIIGV IAAAVWSMWG KKDAGTELTN YQTLATNTIG
Escherichia coli ETEC CofA (CEJ09700.1) VLGIIGTIAA GVVILAQRAF DSRTVSELVT NTNTIRVAMK
Escherichia coli ETEC LngA (CRZ21511.1) VLGIIGTIAA GVVILAQRAF DSRAVTDLVT NTNTVRVAMK
Vibrio cholerae TcpA (1OQV) VLGIMGVVSA GVVTLAQRAI DSQNMTKAAQ SLNSIQVALT
Escherichia coli BfpA (WP_000253757.1) VLALAATVTA GVMFYYQSAS DSNKSQNAIS EVMSATSAIN
Pseudomonas aeruginosa Flp (WP_703141191) -------MKN LTLLVYCK-- -----VRAFL ADEEGANAIE
Pseudomonas aeruginosa PAO1 Flp-1 (NP_252996.1) -------MKN LTLFVYCK-- -----VRAFL ADEEGANAIE
Actinobacillus actinomycetemcomitans Flp-1 (AAK00326.1) ---------M LNTLTTKAYI KASEAIRSFR ENQAGVTAIE
Actinobacillus actinomycetemcomitans Flp-2 (AAK00327.1) ---------M MDLLDY-FYR QVVFFSCNFY RNRQGITSVE
Caulobacter crecentus PilA (AAF40189.1) ---------- -----MTKFV TR------FL KDESGATAIE

	81
Eikenella corrodens EcpA (CAA78250.1)	---------- ---------- -AALFEGRTP VLAATAAAG-
Moraxella bovis PilE (AAA53087)	---------- ---------- -AALFEGKTP VVNPSADGIA
Moraxella bovis TfpQ (AAA25308.1)	---------- --------AA L---FEGKTP KLGKAANDTE
Neisseria gonorrhoeae PilE (CAA47349.1)	---------- ---------- EYYLNNGIWP ENNASAGVAS
Neisseria meningitidis PilE (CAA73472.1)	---------- ---------- EYYLNNGEWP EDNDKAGVA-
Dichelobacter nodosus FimA (AML81112.1)	---------- ---------- -ENLQDGECK GPDADPQSGV
Pseudomonas aeruginosa PAO1 PilA (AAG07913.1)	---------- --------ES LSRGIAGSKI KIGTTASTAT
Vibrio cholerae MshA (AVK79096.1)	-----ISYGR AAIKGIETLD YTAYTDANTP GTVIDGIAH-
Haemophilus influenza PilA (AAX12396.1)	---------- ---------- -LCVYSTGKP SSCSGGSNG-
Actinobacillus actinomycetemcomitans PilA (AAM88344.1)	---------- --------LC I---YNTGKA ENCSGGQNG-
Thermus thermophilus PilA4 (AAM55486.1)	---------- ---------M YQIDNNSYTS NQAALISLG-
Escherichia coli / R721 PilS (WP_000095048.1)	-SLKFQGRYT DSN-----YI KTLYAQGLLP SDMIADTTG-
Escherichia coli / TP114 PilS (ASS85337.1)	-SLKFQGRYT DSN-----YI KTLYAQGLLP SDMIADTTG-
Pseudomonas aeruginosa PA14 (PAPI-1) PilS2 (AAP84205.1)	-ALKTSSGYG --------SS GTNLIPSLIA INGVPKNMSV
Salmonella typhimurium / R64 PilS (BAB91687.1)	SLLKGSDGYT FTSSAKM-TG ALIQMGVIPS GMTVQGDKTS
Escherichia coli / CoIIb-P9 PilS (BAA31138.1)	SLLKGSDGYT FTSSAKM-TG ALIQMGVIPS GMTVQGDKTS
Salmonella Typhi PilS (AAC98887.1)	-MMKGVDGYA FTSGAK--MT DTLIQAGAAK GMTVSGDPAS
Escherichia coli ETEC CofA (CEJ09700.1)	DAYQRDGKYP DYADPLALTA DTIKTGNSTI PVAQLVQLGK
Escherichia coli ETEC LngA (CRZ21511.1)	DAYQRDGVYP DYVSPLNLTS DTIKTDTGKI PAAQLVQLGK
Vibrio cholerae TcpA (1OQV)	QTYRGLGNYP A-------TA DATAASKLTS GLVSLGKIS-
Escherichia coli BfpA (WP_000253757.1)	GLYIGQTSYS G-------LD STILLNTSAI PDNYKDTTN-
Pseudomonas aeruginosa Flp (WP_703141191)	---------- ---------- ---------- ----------
Pseudomonas aeruginosa PAO1 Flp-1 (NP_252996.1)	---------- ---------- ---------- ----------
Actinobacillus actinomycetemcomitans Flp-1 (AAK00326.1)	---------- ---------- ---------- ----------
Actinobacillus actinomycetemcomitans Flp-2 (AAK00327.1)	---------- ---------- ---------- ----------
Caulobacter crecentus PilA (AAF40189.1)	---------- ---------- ---------- ----------

	121
Eikenella corrodens EcpA (CAA78250.1)	AAATPPNEWV GMLDNPTSNL LSA------- --ATLTPGAN
Moraxella bovis PilE (AAA53087)	EVDLGLGEAA TPRSNLLSAV SSTFT----- --KGKSNGTI
Moraxella bovis TfpQ (AAA25308.1)	EDI------- -GLTTTGGTA RSNLMSS--- --VNIGGGAF
Neisseria gonorrhoeae PilE (CAA47349.1)	SAADIKGKYV KSVTVANGVV TATMLS---- --SGVNNEIK
Neisseria meningitidis PilE (CAA73472.1)	SASDIKGKYV KSVTVANGVV TATMLS---- --SGVNNEIK
Dichelobacter nodosus FimA (AML81112.1)	VGNEDKGKYA LAKIDGTYNE SETD------ --AGKPNGCK
Pseudomonas aeruginosa PAO1 PilA (AAG07913.1)	ETYVGVEPDA NKLGVI---- ---------- --AVAIEDSG
Vibrio cholerae MshA (AVK79096.1)	--KFGYPTAV NDSTANLGGI MQTLDTSSEF IALQSSTGTN
Haemophilus influenza PilA (AAX12396.1)	--------IA ADITTAKG-- ---------- --YVKSVTTS
Actinobacillus actinomycetemcomitans PilA (AAM88344.1)	---------- ---------I QADVADASKQ K-YVKSTAVK
Thermus thermophilus PilA4 (AAM55486.1)	---------- --LKSTPANV TFSIVSASAN S-YCMIAGHS
Escherichia coli / R721 PilS (WP_000095048.1)	--ASAKNPWG GSVTITTSSD KYSFNVV--- --EANVPQKN
Escherichia coli / TP114 PilS (ASS85337.1)	--ASAKNPWG GSVTITTSSD KYSFNVV--- --EASVPKKN
Pseudomonas aeruginosa PA14 (PAPI-1) PilS2 (AAP84205.1)	SSGVVYNVYG GSVTVSSTGM GFSIT----- --TSKLPQDA
Salmonella typhimurium / R64 PilS (BAB91687.1)	GTATLYNAWG GAVTVAPAST SGFNNGFTVT --YDKVPQDA
Escherichia coli / CoIIb-P9 PilS (BAA31138.1)	GTATLYNAWG GAVTVAPAST SGFNNGFTVT --YDKVPQDA
Salmonella Typhi PilS (AAC98887.1)	GSATLWNSWG GQIVVAPDTA GGTGFNNGFT ITTNKVPQSA
Escherichia coli ETEC CofA (CEJ09700.1)	LTPDEARNGI SGDYIGIGGA KTSNSNVNKG F-VIELNGLS
Escherichia coli ETEC LngA (CRZ21511.1)	ITPDEVRNNI SGDFIAIGGA LTSDGATVKK GFAIELNGLS
Vibrio cholerae TcpA (1OQV)	-SDEAKNPFI GTNMNIFSFP RNAAANKAF- --AISVDGLT
Escherichia coli BfpA (WP_000253757.1)	--KKITNPFG GELNVGPANN NTAFGYYLT- --LTRLDKAA
Pseudomonas aeruginosa Flp (WP_703141191)	---------- ---------- ---------- --YAVIAGLI
Pseudomonas aeruginosa PAO1 Flp-1 (NP_252996.1)	---------- ---------- ---------- --YAVIAGLI
Actinobacillus actinomycetemcomitans Flp-1 (AAK00326.1)	---------- ---------- ---------- --YGLIAIAV
Actinobacillus actinomycetemcomitans Flp-2 (AAK00327.1)	---------- ---------- ---------- --YGLIAVAI
Caulobacter crecentus PilA (AAF40189.1)	---------- ---------- ---------- --YGLIVALI

	161			
Eikenella corrodens EcpA (CAA78250.1)	AGDVTFVGTL	-G--------	--ENAN----	-SSIHGATIT
Moraxella bovis PilE (AAA53087)	SGTIG-----	----------	--GNAN----	-NDIHGTVIS
Moraxella bovis TfpQ (AAA25308.1)	ATGAGTLEAT	LG--------	--NRAN----	-KDIAGAVIT
Neisseria gonorrhoeae PilE (CAA47349.1)	GKKLSLWAXR	Q---------	----------	-DGSVKWFCG
Neisseria meningitidis PilE (CAA73472.1)	GKKLSLWAKR	Q---------	----------	-DGSVKWFCG
Dichelobacter nodosus FimA (AML81112.1)	VEIAYGQGTA	----------	----------	-EGKISKLIT
Pseudomonas aeruginosa PAO1 PilA (AAG07913.1)	AGDITFTFQT	----------	--GTSS----	-PKNATKVIT
Vibrio cholerae MshA (AVK79096.1)	SNQANLWIDI	-G--------	--FNGY----	-NDLCVRYTA
Haemophilus influenza PilA (AAX12396.1)	NGAITVKG--	----------	--DGTL----	-ANMEYILQA
Actinobacillus actinomycetemcomitans PilA (AAM88344.1)	GGVITVTG--	----------	--KGSL----	-DKISYTLTA
Thermus thermophilus PilA4 (AAM55486.1)	GGTV--WFAA	----------	----------	-TPDKGVYKT
Escherichia coli / R721 PilS (WP_000095048.1)	CMAMVNALRS	----------	----------	-SSAISKINN
Escherichia coli / TP114 PilS (ASS85337.1)	CMAMVNALRS	----------	----------	-SSAISKINN
Pseudomonas aeruginosa PA14 (PAPI-1) PilS2 (AAP84205.1)	CITLATKIAK	NT--------	--FEQT----	-KINSGSSIT
Salmonella typhimurium / R64 PilS (BAB91687.1)	CIQIATRISK	TGL-------	--TNGI----	-TLNSTAHSD
Escherichia coli / ColIb-P9 PilS (BAA31138.1)	CIQIATRISK	TGL-------	--TNGI----	-TLNSTAHSD
Salmonella Typhi PilS (AAC98887.1)	CVSISTGMSR	SG--------	-GTSGI----	-KINGNNHTD
Escherichia coli ETEC CofA (CEJ09700.1)	QEQCRSILGQ	VGNNWEFVEV	GTSGSG----	-SYSITAQVD
Escherichia coli ETEC LngA (CRZ21511.1)	QEQCRSILGQ	VGNNWEYVAV	GNSASG----	-SYAMQTAGV
Vibrio cholerae TcpA (1OQV)	QAQCKTLITS	VGDMFPYIAI	KAGGAVALAD	LGDFENSAAA
Escherichia coli BfpA (WP_000253757.1)	CVSLATLNLG	TSA-------	--KGYGVNIS	GENNITSFGN
Pseudomonas aeruginosa Flp (WP_703141191)	AVALIAVLSP	TD--------	--SGIV----	-GGLKAFFDG
Pseudomonas aeruginosa PAO1 Flp-1 (NP_252996.1)	AVALIAVLSP	TD--------	--SGIV----	-GGLKAFFDG
Actinobacillus actinomycetemcomitans Flp-1 (AAK00326.1)	AVLIVAVFYS	N---------	--NGFI----	-ANLQSKFNS
Actinobacillus actinomycetemcomitans Flp-2 (AAK00327.1)	AVFVVAVLVG	D---------	--NSFI----	-KALSGKFSD
Caulobacter crecentus PilA (AAF40189.1)	AVVIVTAVTT	LG--------	--TNLR----	-TAFTKAGAA

	201			
Eikenella corrodens EcpA (CAA78250.1)	LTCTASGE-W	TCAVAAGTAT	GWKTKFVPSG	CN*-------
Moraxella bovis PilE (AAA53087)	QERDATGV-W	SCKVTGNGT-	GWKDKFIPTG	CTKA*-----
Moraxella bovis TfpQ (AAA25308.1)	QSRDAEGV-W	TCTINGSAAP	GWKSKFVPTG	CKE*------
Neisseria gonorrhoeae PilE (CAA47349.1)	QPVTRTGDND	DTVADAKDGK	EIDTKHLPST	CRDTSSAGK*
Neisseria meningitidis PilE (CAA73472.1)	QPVTRTGDND	DTVADAKDGK	EIDTKHLPST	CRDNFDAS*-
Dichelobacter nodosus FimA (AML81112.1)	GKKLVLDQLV	NGSFIAGDGT	DLADKFIPNA	VKAKK*----
Pseudomonas aeruginosa PAO1 PilA (AAG07913.1)	LNRTADGV-W	ACKSTQDPM-	-----FTPKG	CDN*------
Vibrio cholerae MshA (AVK79096.1)	ATSNNPATVA	MQTGVAGTAC	GKPVP*----	----------
Haemophilus influenza PilA (AAX12396.1)	SGNAATGVTW	TTTCKGTDAS	LFPANFCGSV	TQ*-------
Actinobacillus actinomycetemcomitans PilA (AAM88344.1)	SGTAATGVSW	NAACGNNAG-	----IFPAGF	CS*-------
Thermus thermophilus PilA4 (AAM55486.1)	NTAVTSSQPE	SCP*------	----------	----------
Escherichia coli / R721 PilS (WP_000095048.1)	TSTSTVSAAT	VCASDSNTLT	FSTDS*----	----------
Escherichia coli / TP114 PilS (ASS85337.1)	TSTSTVDAAT	VCSSDSNTLT	FSTDS*----	----------
Pseudomonas aeruginosa PA14 (PAPI-1) PilS2 (AAP84205.1)	GEVTTAAATQ	ACSSDSNSIT	WTYSS*----	----------
Salmonella typhimurium / R64 PilS (BAB91687.1)	GKVTTEEAST	QCKADNGSTG	TNKLIFTING	*---------
Escherichia coli / ColIb-P9 PilS (BAA31138.1)	GKVTTEEAST	QCKADNGSTG	TNKLIFTING	*---------
Salmonella Typhi PilS (AAC98887.1)	AKVTAEIASS	ECTADNGRTG	TNTLVFNYNG	*---------
Escherichia coli ETEC CofA (CEJ09700.1)	MTKESGNNTT	LRSLGLNGQN	SITSSSILNT	CTATVNSIIL
Escherichia coli ETEC LngA (CRZ21511.1)	NMSVAATGNI	LRTLGTDGQK	TLTAERILGT	CTATVNSITL
Vibrio cholerae TcpA (1OQV)	AETTGVGVIK	SIAPASKNLD	LTNITHVEKL	CKGTAPFGVA
Escherichia coli BfpA (WP_000253757.1)	SADQAAKSTA	ITPAEAATAC	KNTDSTNKVT	YFMK*-----
Pseudomonas aeruginosa Flp (WP_703141191)	VGTKVGGLAP	D*--------	----------	----------
Pseudomonas aeruginosa PAO1 Flp-1 (NP_252996.1)	VGEKVGGLAP	TAN*------	----------	----------
Actinobacillus actinomycetemcomitans Flp-1 (AAK00326.1)	LASTVASANV	TK*-------	----------	----------
Actinobacillus actinomycetemcomitans Flp-2 (AAK00327.1)	LTTTVSGAIV	SKSS*-----	----------	----------
Caulobacter crecentus PilA (AAF40189.1)	VSTAAGT*--	----------	----------	----------

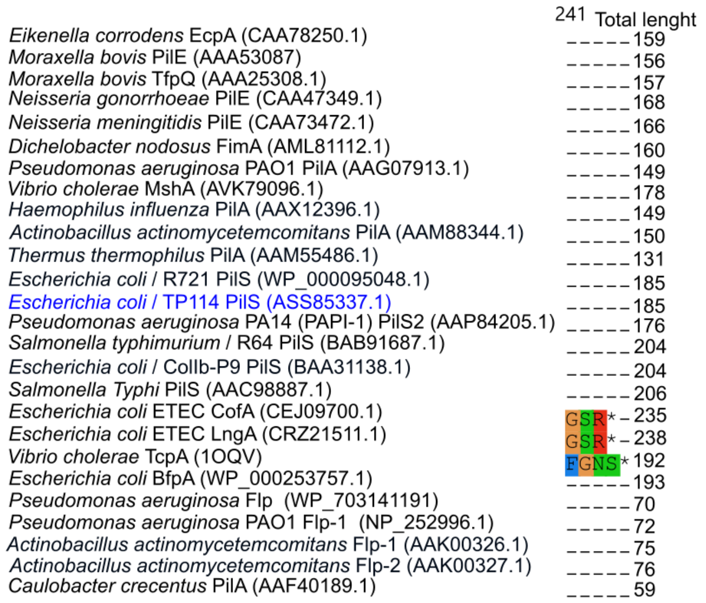

Supplementary Figure S2. Major pilin sequences alignment. The selected major pilin sequences used for the phylogenetic tree were aligned using the MUSCLE option in SeaView 5.0.4 software. The predicted cleavage site is indicated by an arrow immediately after the conserved glycine. The conserved glutamate at position 5 of the mature pilins is indicated in a box. TP114 PilS is highlighted in blue.

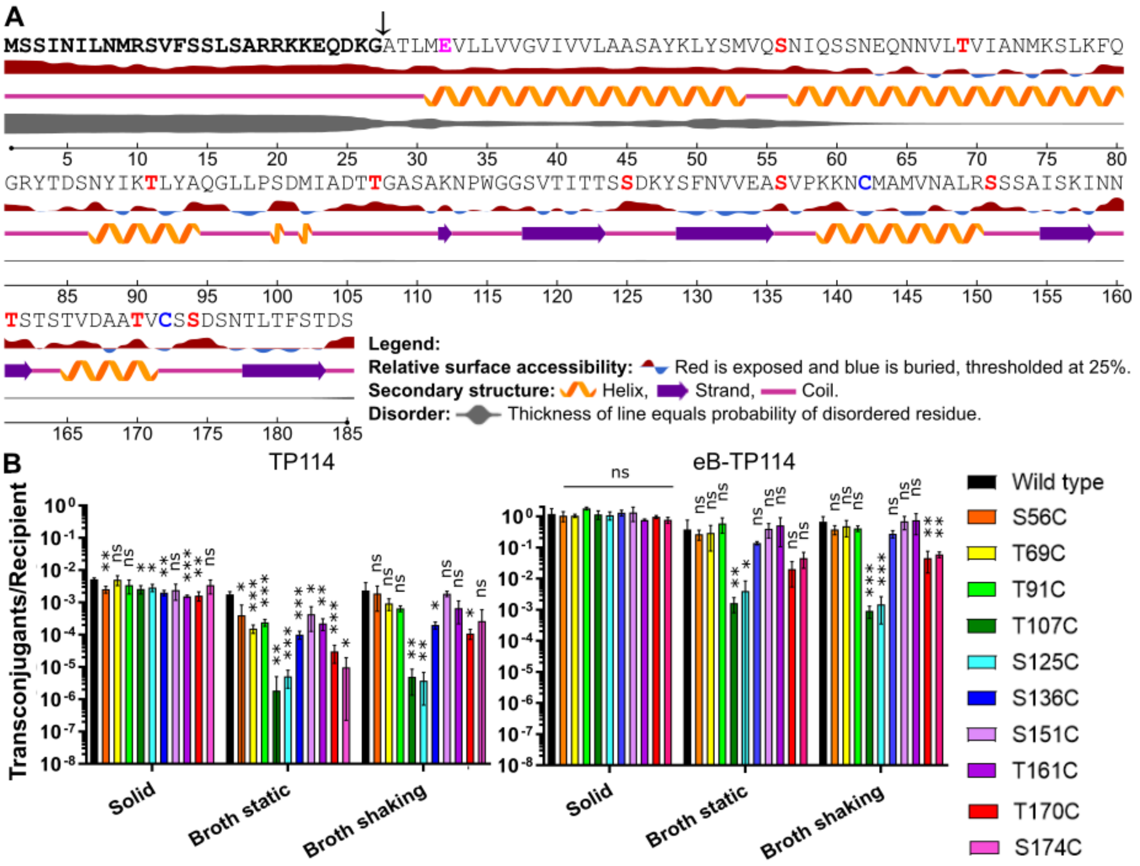

Supplementary Figure S3. Cysteine knock-in positions in PilS and their effects on conjugation frequencies. (A) Illustration of the predicted topology (surface accessibility and secondary structure) of amino acids constituting PilS according to NetSurfP-2.0 server. The putative class III signal peptide is in bold, and its predicted cleavage site is indicated by an arrow immediately after the conserved glycine. The conserved glutamate at position 5 of the mature pilin is indicated in purple. The two natural cysteines already present in PilS sequence and potentially implicated in disulphide bonds for stabilization are indicated in bold blue, while the serine and threonine residues, where cysteine knock-in have been introduced individually, are shown in bold red. (B) Conjugation frequencies of each PilS single cysteine knock-in mutant in TP114 or the derepressed eB-TP114 derivative compared to the wild type plasmids in different conditions. Bars and error bars indicate the mean and standard deviation calculated from biological triplicate, respectively. One-way ANOVA statistical analysis results are shown as: ns $P \geq 0.05$, * $P < 0.05$, ** $P \leq 0.01$, *** $P \leq 0.001$.

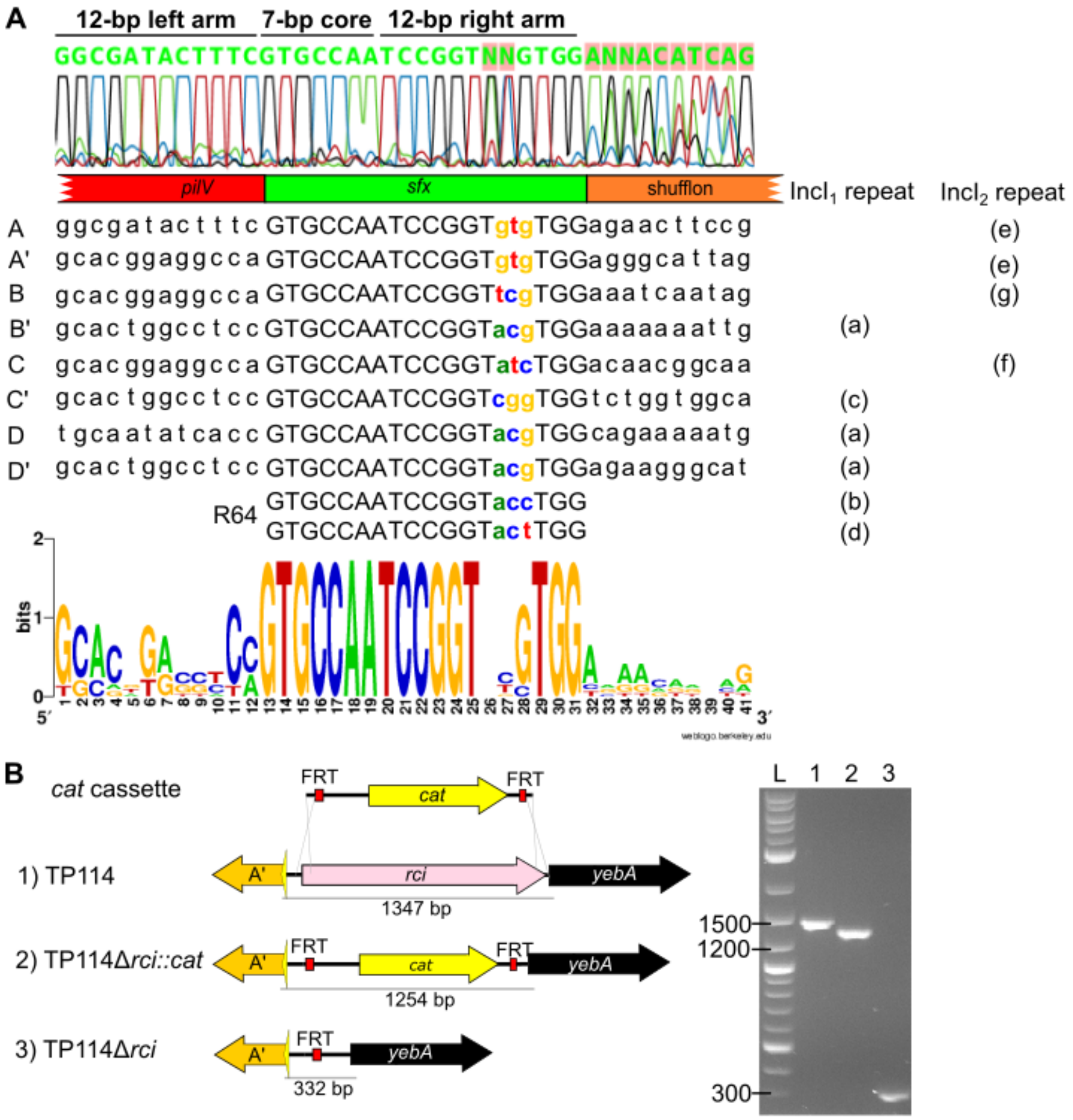

Supplementary Figure S4 Analysis of *sfx* repeat regions and deletion of shufflase *rci* gene in TP114. (A) Multiple alignments of the eight *sfx* repeat regions of TP114 shufflon according to the gene order depicted in figure 3 and the corresponding consensus sequence. The variable nucleotides are shown in lowercase and colour for the 3-bp variable region. Four out of eight conserved core and right arm sequences are identical to the sequences found in IncI1 plasmid R64 (a and c) or IncI2 plasmid R721 (e and f). The IncI2 repeat sequences found in R721 as well as the newly identified *sfx* sequence were designed as repeat e to g. The eight *sfx* repeat regions were analyzed by the

sequence logo program. (B) Schematic representation of the steps to remove the shufflase gene (*rci*) in TP114 by recombineering, and corresponding PCR confirmations. The *cat* cassette was amplified by PCR from pKD3 plasmid. All DNA fragments are drawn to scale. Amplified DNA regions are indicated by gray lines. L: 1 kb Plus DNA Ladder (NEB).

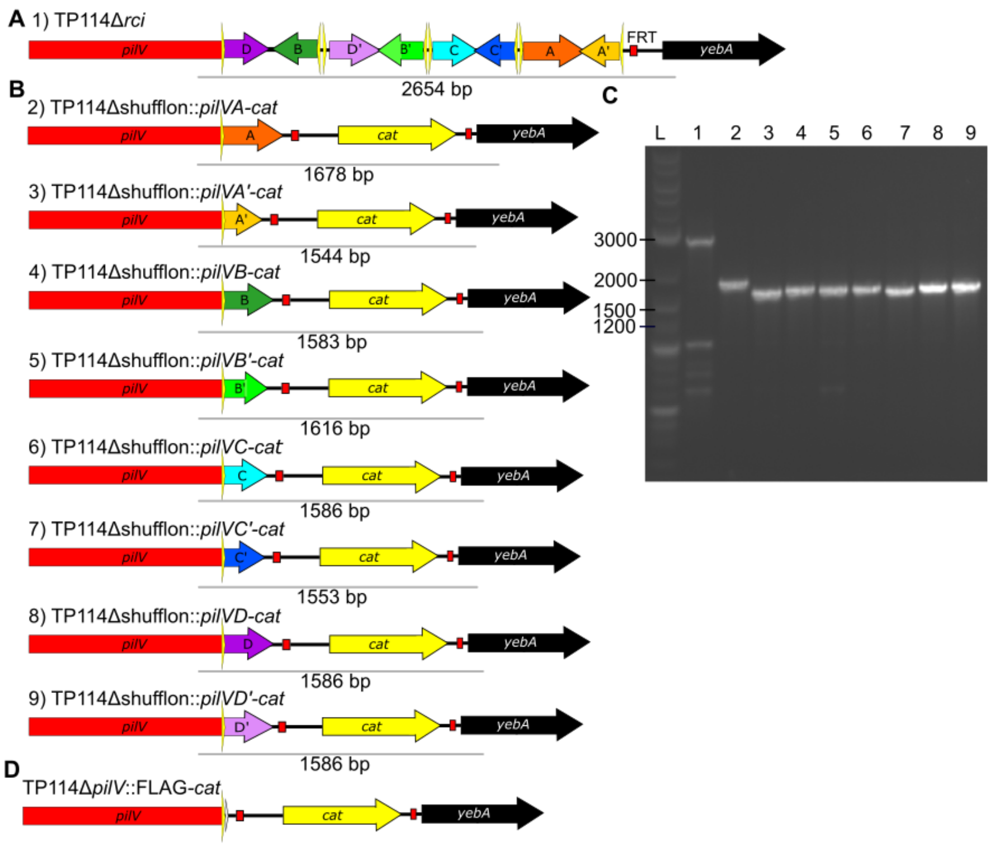

Supplementary Figure S5 Representation of TP114 derivatives. (A) Schematic illustration of the TP114 shufflon conformation resulting from the deletion of the shufflase gene (*rci*) (see Supplementary Figure S3B), in which the D variant was fixed at the C-terminal of *pilV*. (B) Representation of the different *pilV* variant derivatives constructed by replacing the entire shufflon by recombineering. Amplified regions for PCR confirmation are indicated by gray lines. All DNA fragments are drawn to scale (C) PCR amplifications confirming each constructed *pilV* variant. L: 1 kb Plus DNA Ladder (NEB). (D) Representation of TP114Δ*pilV*::FLAG-*cat*.

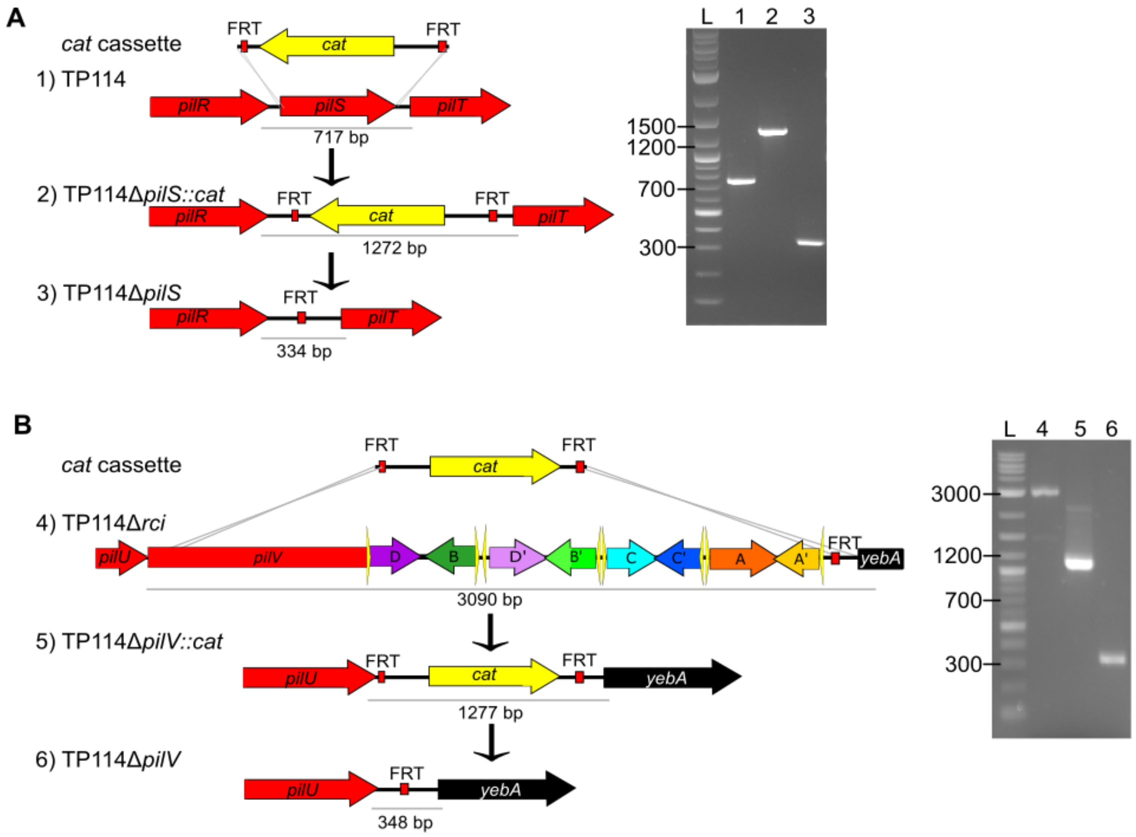

Supplementary Figure S6. Deletion of TP114 major (*pilS*) and minor (*pilV*) pilins. Schematic illustration of the steps to remove individually the major pilin (A) or the minor pilin along with the entire shufflon (B) by recombineering. PCR confirmations of the resulting constructs are also shown. The *cat* cassette was amplified by PCR from pKD3 plasmid. All DNA fragments are drawn to scale. Amplified DNA regions for PCR confirmations are indicated by gray lines. L: 1 kb Plus DNA Ladder (NEB).

Supplementary References.

1. Ceccarelli D, Daccord A, René M, Burrus V. 2008. Identification of the Origin of transfer (*oriT*) and a new gene required for mobilization of the SXT / R391 family of integrating conjugative elements. J Bacteriol 190:5328–5338.
2. Cress BF, Linhardt RJ, Koffas MAG. 2013. Draft genome sequence of *Escherichia coli* strain Nissle 1917 (Serovar O6:K5:H1). Genome Announc 1:e00047-13.
3. Neil K, Allard N, Grenier F, Burrus V, Rodrigue S. 2020. Highly efficient gene transfer in the mouse gut microbiota is enabled by the Incl2 conjugative plasmid TP114. Commun Biol 3:523.
4. Baba T, Ara T, Hasegawa M, Takai Y, Okumura Y, Baba M, Datsenko KA, Tomita M, Wanner BL, Mori H. 2006. Construction of *Escherichia coli* K-12 in-frame, single-gene knockout mutants: The Keio collection. Mol Syst Biol 2:2006.0008.
5. Poulin-Laprade D, Brouard JS, Gagnon N, Turcotte A, Langlois A, Matte JJ, Carrillo CD, Zaheer R, McAllister TA, Topp E, Talbot G. 2021. Resistance determinants and their genetic context in Enterobacteria from a longitudinal study of pigs reared under various husbandry conditions. Appl Environ Microbiol 87:1–20.
6. Guzman LM, Belin D, Carson MJ, Beckwith J. 1995. Tight regulation, modulation, and high-level expression by vectors containing the arabinose P(BAD) promoter. J Bacteriol 177:4121–4130.
7. Datta S, Costantino N, Court DL. 2006. A set of recombineering plasmids for gram-negative bacteria. Gene 379:109–115.
8. Huguet KT, Rivard N, Garneau D, Palanee J, Burrus V. 2020. Replication of the *Salmonella* Genomic Island 1 (SGI1) triggered by helper IncC conjugative plasmids promotes incompatibility and plasmid loss. PLoS Genet 16(8): e1008965.
